# Investigation of DDT resistance mechanisms in *Anopheles funestus* populations from northern and southern Benin reveals a key role of the *GSTe2* gene

**DOI:** 10.1186/s12936-020-03503-2

**Published:** 2020-12-17

**Authors:** Genevieve M. Tchigossou, Seun M. Atoyebi, Romaric Akoton, Eric Tossou, Djegbe Innocent, Jacob Riveron, Helen Irving, Akadiri Yessoufou, Charles Wondji, Rousseau Djouaka

**Affiliations:** 1 International Institute of Tropical Agriculture, Cotonou, 08 BP 0932 Benin; 2 University of Abomey Calavi, BP 526, Cotonou, Benin; 3 Cell Biology and Genetics Unit, Department of Zoology, University of Ibadan, Oyo, Oya State Nigeria; 4 Technologies, Engineering and Mathematics, National University of Sciences, Ecole Normale Supérieure de Natitingou, Natitingou, BP 123 Benin; 5 Liverpool School of Tropical Medicine, Pembroke PlaceLiverpool, L3 5QA UK; 6 Center for Research in Infectious Diseases (CRID), Yaoundé, Centre Region Cameroon

**Keywords:** *Anopheles funestus*, Mechanism of resistance, DDT, *GSTe2*, Benin

## Abstract

**Background:**

Understanding the molecular basis of insecticide resistance in mosquito, such as *Anopheles funestus,* is an important step in developing strategies to mitigate the resistance problem. This study aims to assess the role of the *GSTe2* gene in DDT resistance and determine the genetic diversity of this gene in *An. funestus*.

**Methods:**

Gene expression analysis was performed using microarrays and PCR while the potential mutation associated with resistance was determined using sequencing.

**Results:**

Low expression level of *GSTe2* gene was recorded in Burkina-Faso samples with a fold change of 3.3 while high expression (FC 35.6) was recorded in southern Benin in Pahou (FC 35.6) and Kpome (FC 13.3). The sequencing of *GSTe2* gene in six localities showed that L119F-*GSTe2* mutation is almost getting fixed in highly DDT-resistant Benin (Pahou, Kpome, Doukonta) and Nigeria (Akaka Remo) mosquitoes with a low mutation rate observed in Tanongou (Benin) and Burkina-Faso mosquitoes.

**Conclusion:**

This study shows the key role of the *GSTe2* gene in DDT resistant *An. funestus* in Benin. Polymorphism analysis of this gene across Benin revealed possible barriers to gene flow, which could impact the design and implementation of resistance management strategies in the country.

## Background

Malaria remains the most severe infectious disease and a major public health challenge in sub-Saharan Africa [1]. The mortality and the loss of productivity due to the illness, has devastating effects on cognitive development in children surviving the disease, leaving many disabled for life [2]. Since the discovery of the connection between *Anopheles* vectors and malarial transmission in 1897, vector control strategies have been the most widely used malarial control measures [3]. These measures (based on insecticide use) are insecticide treated bed-nets (ITN) and indoor residual spraying (IRS), both of which have been shown to be effective for reducing malaria prevalence in Africa [4]. One of the insecticides of choice for IRS is DDT (dichloro-diphenyl-trichloro-ethane) because of its high insecticidal activity, low acute mammalian toxicity, wide spectrum use, low price, and long duration of activity.

The availability of dichlorodiphenyltrichloroethane (DDT) and other insecticides in the 1940s marked a new era for malarial control in the world. The effectiveness of DDT against indoor resting mosquitoes led to the adoption of the Global Eradication Programme of Malaria in 1955, coordinated and supported by the World Health Organization (WHO). Although the use of DDT raises concerns of potential harm to the environment and human health, mainly because of the persistent and bio-accumulative nature of DDT and its potential to magnify through the food chain, it continued to be used for pest control, for which exemptions were granted by the federal government and it is still available for public health use today [5].

Inevitably, the major malaria vectors, *Anopheles gambiae* and *Anopheles funestus*, have developed resistance to this insecticide. The basic mechanisms underlying insecticide resistance include insecticide target-site mutations, and increased metabolic detoxification of the insecticide through overproduction or elevated enzymatic activity [6]. Three enzyme families are primarily involved in insecticide detoxification: the carboxylesterases (COEs), glutathione-S-transferases (GSTs) and cytochrome P450s (P450s). DDT resistance in *An. gambiae* can be due either to a specific detoxification mechanism (glutathione-S-transferase) or to a nerve insensitivity resulting from a modification of the target site (sodium channel). The latter, governed by the *kdr* gene, reduces both the knockdown and lethal effects of DDT [7]. In West Africa, it induces a cross-resistance to pyrethroids, which also depends on *kdr* mutation [7, 8]. In contrary, no *kdr* mutation has been detected in *An. funestus* so far [9–11]. Indeed, a single amino acid change in the binding pocket of the glutathione-S-transferase epsilon 2 (*GSTe2*) gene, coupled with increased transcription of this gene, confers a high level of DDT resistance and also cross-resistance to pyrethroids in *An. funestus*. Furthermore, analysis of *GSTe2* polymorphism established that the L119F-Gste2 mutation is tightly associated with metabolic resistance to DDT and its geographical distribution strongly correlates with DDT resistance patterns across Africa [12]. Nevertheless, the strong contrast in the allele frequencies of the L119F-GSTe2 frequencies despite the similar resistance profile recorded in *An. funestus* populations from two localities in Ghana [13] suggest that possible barriers to gene flow could exist between populations of the same country. Such differences in the underlying resistance mechanisms should be taken into account when designing suitable insecticide resistance management strategies. In southern Benin (Kpome and Pahou), *An. funestus* was found to be highly resistant to DDT [14] [15] while the population from Tanongou was moderately resistant to DDT with 90% mortality [16]. Also, as *GSTe2* gene has been associated with DDT resistance patterns across Africa, this study aimed to investigate the role of the *GSTe2* gene in DDT resistance across Benin to fill the knowledge gap by checking if this resistance is driven by the same mechanism.

## Methods

### Samples description

In this study, mosquitoes from the previously published research results were used to further describe the molecular basis of DDT resistance in *An. funestus* population from different localities [12, 14–16]. Mosquito samples generated from the previous investigation were used for genetic analysis in this work.

### Study area and mosquito collection

**Fig. 1:**
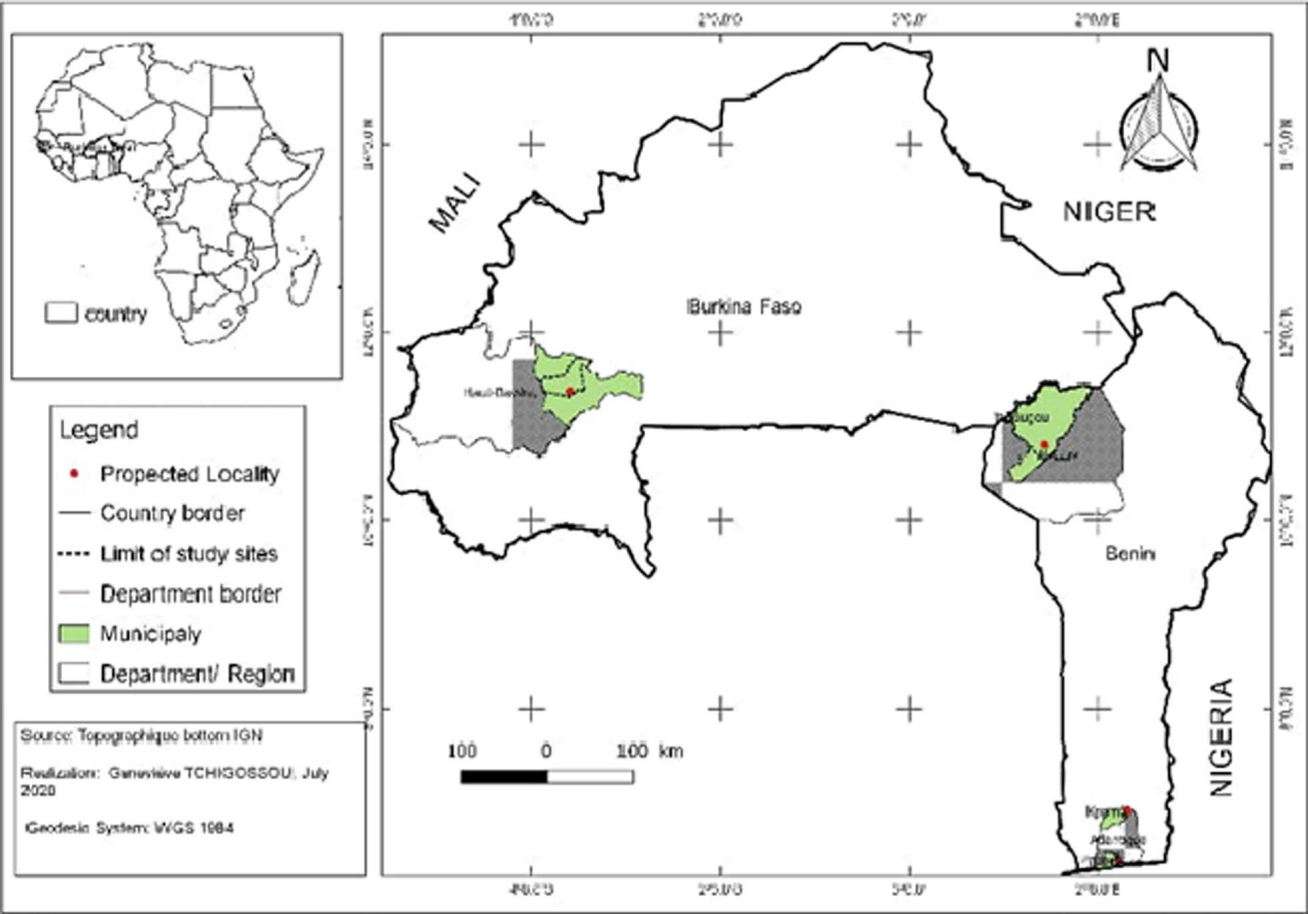
Map showing the study sites

Adult anopheles mosquitoes were collected from three (3) locations in Benin: Kpome in South-Est (6° 55′ N, 2° 19′E), Pahou (6° 23′ N, 2° 13′E) in South-West and Tanongou in North West (10° 48′ N, 1° 26′ E). Mosquitoes were also collected in South-West Burkina Faso (11° 23′ N, 4° 24′ E) (Fig. 1). The selected sites are located in close proximity with rivers, swamps as these permanent water bodies are suitable breeding sites for *An. funestus*. After obtaining consent from village chiefs and house owners, indoor resting mosquitoes were collected from December 2013 to March 2014 inside households using electric aspirator. Blood fed mosquitoes collected were kept in cups until fully gravid before being subjected to the forced-egg laying technique [17]. The eggs obtained were pooled and reared in a mineral water. Larvae were reared under standard insectary conditions (26 ± 2 °C with a relative humidity of 80%) and were fed daily with Tetramin™ baby fish food. The water of each larvae bowl was changed every two days to reduce mortality. F1 adult generated were pooled in cages for subsequent analyses.

### Microarrays

A custom microarray chip containing 44,000 probes (4 × 44 k) [18] was used to identify the set of genes associated with DDT resistance in Pahou and Burkina-Faso. The 8 × 60 k (60 mer) Agilent *An. funestus* chip was used to screen for the genes involved in resistance of *An. funestus* from Kpome. This Agilent microarray chip was designed using the eArray program (Agilent, Santa Clara, CA, USA) (A-MEXP-2374) by adding the 15,527 expressed sequence tags (ESTs) generated from another transcriptome sequencing of *An. funestus* [19] to the previous 4 × 44 k array (A-MEXP-2245) [18]. Labelled cRNA was obtained from three biological replicates (10 mosquitoes per replicate) for the following samples: (i) resistant (R) (mosquitoes alive after a 1-h exposure to 4% DDT); (ii) control (C) (mosquitoes unexposed to insecticide and thus representative of the wild-type population); and (iii) susceptible (S) (unexposed mosquitoes from the fully susceptible laboratory strain of *An. funestus*: FANG) making a total of 60 mosquitoes per locality (Pahou, Kpome and Burkina-Faso). Complementary RNA (cRNA) was amplified from each sample using the Agilent Quick Amp Labelling Kit (two-colour) following the manufacturer’s protocol. These cRNA were reciprocally hybridized against each other comparing R-S for resistant *vs.* susceptible, C-S for control *vs.* susceptible. Microarray data were analyzed using Genespring GX 13.0 software. To identify differentially expressed genes, a cut-off of twofold-change (FC) and a statistical significance of P < 0.05 using Storey with boostrapping correction for multiple testing were applied. These results were compared to those obtained from Kpome [20].

### Quantitative reverse transcriptase PCR

Three genes (*GSTe2*, *CYP6P9a* and *CYP6P9b)* (Table 1) up-regulated from the microarray analysis and mostly associated with DDT and pyrethroids resistance [12, 14, 18, 21] were assessed by qRT-PCR to validate their expression pattern using the three biological replicates for resistant, control, and FANG. cDNA from the Resistant (R), Control (C) and FANG (S) populations were synthesized using one microgram of total RNA from each of the three biological replicates. The relative expression level and FC of each target gene in R and C relative to S were calculated according to the 2-ΔΔCT method incorporating the PCR efficiency [22] after normalization with the housekeeping genes ribosomal protein S7 (*RSP7*; AFUN007153-RA), and actin (*Actin*; AFUN006819) (Table 1). The results were compared to those obtained in Kpome.

**Table 1:** List of top upregulated detoxification gene in *Anopheles funestus* from Pahou and Burkina-Faso exposed and unexposed to DDT

Probe name	Systematic name	Burkina-Faso	Pahou		Description
		Rddt-S	Rddt-S	C-S	
CUST_1822_PI406199769	combined_c920 (Gste2)	3.3	35.6	11.9	Glutathione-s-transferase gst
CUST_9502_PI406199769	combined_c4812	3.0	2.8	2.1	Short-chain dehydrogenase
CUST_2953_PI406199769	combined_c1489	2.1	3.6	3.0	Argininosuccinate lyase
CUST_111_PI406199775	CYP4D26	2.0	2.1	3.3	Cytochrome p450
CUST_13272_PI406199769	combined_c6791	6.6	2.6		Cytochrome p450
CUST_2464_PI406199772	CD578169.1	2.4	3.7		Trypsin
CUST_15002_PI406201128	AGAP007662-RA___2L	2.2	4.7		Short-chain dehydrogenase
CUST_15001_PI406201128	AGAP007662-RA	2.2	6.1		Short-chain dehydrogenase
CUST_48_PI406199775	CYP6z3	2.1	2.5		Cytochrome p450
CUST_3754_PI406199772	CD577506.1	3.4		4.1	Cuticle protein
CUST_639_PI406199788	gb-GSTS1_2	3.4		3.6	Glutathione s-transferase
CUST_7429_PI406199769	combined_c3760	2.8		3.2	Chymotrypsin 1
CUST_7428_PI406199769	combined_c3760	2.4		3.5	Chymotrypsin 1
CUST_1963_PI406199772	CD664227.1	2.2		2.5	Alcohol dehydrogenase
CUST_633_PI406199788	gb-GSTO1	2.1		2.1	Glutathione s-transferase
CUST_102_PI406199788	gb-COE15O	2.1		3.2	Carboxylesterase
CUST_4649_PI406199798	AGAP003343-RA___2R	2.1		2.1	Cytochrome p450
CUST_4923_PI406199772	BU038981	2.0		2.1	Alcohol dehydrogenase
CUST_483_PI406199788	gb-CYP6Z4	2.0		2.2	Cytochrome p450
CUST_3109_PI406199772	CD577844.1		5.0	2.0	Cuticle protein
CUST_3835_PI406199772	CD577459.1		4.7	2.4	Cuticle protein
CUST_9503_PI406199769	combined_c4812		3.2	2.5	Short-chain dehydrogenase
CUST_14376_PI406199769	combined_c7513		2.6	3.5	Glutathione transferase
CUST_345_PI406199788	gb-CYP4D17		2.5	3.1	Cytochrome p450
CUST_2520_PI406199772	CD578141.1		2.5	2.2	Short-chain dehydrogenase
CUST_2550_PI406199769	combined_c1287		2.3	2.8	Aldehyde dehydrogenase
CUST_2551_PI406199769	combined_c1287		2.3	3.5	Aldehyde dehydrogenase
CUST_7029_PI406199769	combined_c3556		2.2	2.3	Cytochrome p450
CUST_2914_PI406199772	CD577943.1		2.2	2.3	Short-chain dehydrogenase
CUST_1090_PI406199798	AGAP000881-RA___X		2.1	2.3	Aldehyde dehydrogenase
CUST_1964_PI406199772	CD664227.1		2.0	2.6	Alcohol dehydrogenase
CUST_5005_PI406199798	AGAP003680-RA___2R	3.7			Abc transporter
CUST_7696_PI406199798	AGAP008141-RA___3R	2.5			Argininosuccinate lyase
CUST_10700_PI406199798	AGAP009850-RA___3R	2.3			Abc transporter
CUST_3946_PI406199772	CD577403.1	2.2			Glutathione s-transferase
CUST_4649_PI406199798	AGAP003343-RA___2R	2.1			Cytochrome p450
CUST_8727_PI406199769	combined_c4419	2.1			Abc transporter
CUST_12208_PI406199769	combined_c6213	2.0			Argininosuccinate lyase
CUST_44_PI406199775	CYP6z1		3.5		Cytochrome p450
CUST_27_PI406199775	CYP6P9a		2.6		Cytochrome p450
CUST_11_PI406199775	CYP6P1		2.1		Cytochrome p450
CUST_13469_PI406199769	combined_c6910		2.1		Glutathione s-transferase e2
CUST_3488_PI406199769	combined_c1762		2.1		Abc transporter
CUST_402_PI406199788	gb-CYP6AA2			3.6	Cytochrome p450
CUST_353_PI406199788	gb-CYP4G17			2.9	Cytochrome p450
CUST_375_PI406199788	gb-CYP4H24			2.9	Cytochrome p450
CUST_3620_PI406199772	CD577573.1			2.8	Glutathione s-transferase
CUST_277_PI406199788	gb-CYP325D1			2.8	Cytochrome p450
CUST_374_PI406199788	gb-CYP4H24			2.8	Cytochrome p450
CUST_386_PI406199788	gb-CYP4J10			2.6	Cytochrome p450
CUST_401_PI406199788	gb-CYP6AA2			2.5	Cytochrome p450
CUST_237_PI406199788	gb-CYP305A3			2.5	Cytochrome p450
CUST_372_PI406199788	gb-CYP4H19			2.3	Cytochrome p450
CUST_431_PI406199788	gb-CYP6M3			2.2	Cytochrome p450
CUST_5107_PI406201128	AGAP002204-RA_CYP325D1			2.1	Cytochrome p450
CUST_407_PI406199788	gb-CYP6AF1/2			2.0	Cytochrome p450
CUST_3938_PI406199772	CD577407.1			2.0	Glutathione s-transferase

### Genotyping of L119F-GSTe2 resistance

The role of the L119F-Gste2 mutation recently shown to play a major role in the DDT resistance was assessed. Field-collected *An. funestus *sensu stricto (s.s.) females from each selected location were genotyped using a Taqman assay [12]. The reaction was performed in a 10-μl final volume containing 1 × SensiMix (Bioline, London, UK), 800 nM of each primer and 200 nM of each probe using an Agilent MX3005P machine. The following cycling conditions were used: 10 min at 95 °C, 40 cycles of 15 s at 92 °C and 1 min at 60 °C. Two probes labelled with fluorochromes FAM and HEX were used. The FAM detected the mutant allele while the HEX detected the wild allele.

### Genetic diversity of GSTe2 across Benin

A full-length *GSTe2* (exons and introns) was amplified from 10 field-collected female mosquitoes from each location using Phusion High-Fidelity DNA Polymerase (Fermentas, Burlington, Ontario, Canada) and the following conditions: 1 cycle at 95 °C for 5 min; 35 cycles of 94 °C for 20 s, 57 °C for 30 s and 72 °C for 60 s; and 1 cycle at 72 °C for 5 min. The PCR products were purified using the QIAquick PCR Purification Kit (Qiagen, Valencia, CA, USA) and subsequently sequenced. The GSTe2- L119F polymorphic position was detected through a manual analysis of sequence traces and sequences alignments were done using BioEdit. Data were exported to the software DnaSp-version 5.10.01 to detect genetic variability of the *GSTe2* gene among the different populations. A maximum likelihood phylogenetic tree for the coding sequences of *GSTe2* in the five localities was constructed using MEGA 5.2 [23]. The best model was firsly assessed and this indicated that the Jukes-Cantor model best describes the *GSTe2* haplotypes. This was then used to generate the maximum likehood tree using MEGA 5.2. In addition, the level of pairwise genetic differentiation between the populations were determined in dnasp 5. 10 using the Kst statistic [24] and the neighbour-joining tree was built using Mega 6.06 [24].

## Results

### Susceptibility profiles to insecticides

The Pahou population (Benin) had previously been described as highly DDT resistant [14] with no mortality 24 h after 1 h of exposure. The WHO bioassays conducted in Kpome [15] indicated that this *An. funestus* population, which is located approximately 100 km from Pahou, was also resistant to DDT, with 9.1 ± 2.5% mortality 24 h after 1 h of exposure to 4% DDT for females. The population from Tanongou was moderately resistant to DDT with 90% mortality [16].

### Genome-wide transcription microarray analysis

**Fig. 2:**
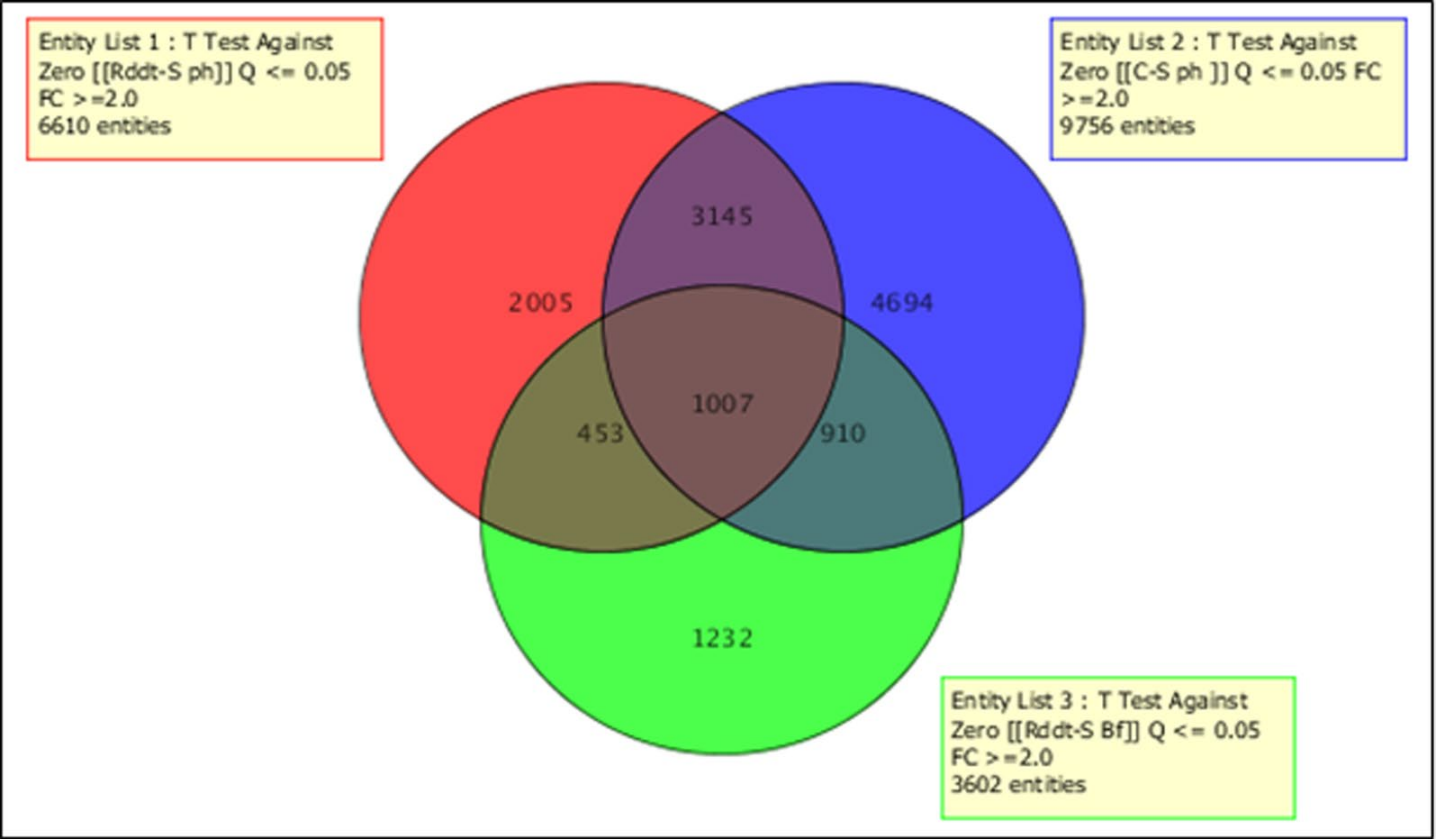
Summary of probes differentially expressed in Pahou and Burkina-Faso samples. The Venn diagrams show the number of probes significantly (P ≤ 0.05) up or down-regulated (FC ≥ 2) in each comparison as well as the commonly expressed probes

**Fig. 3:**
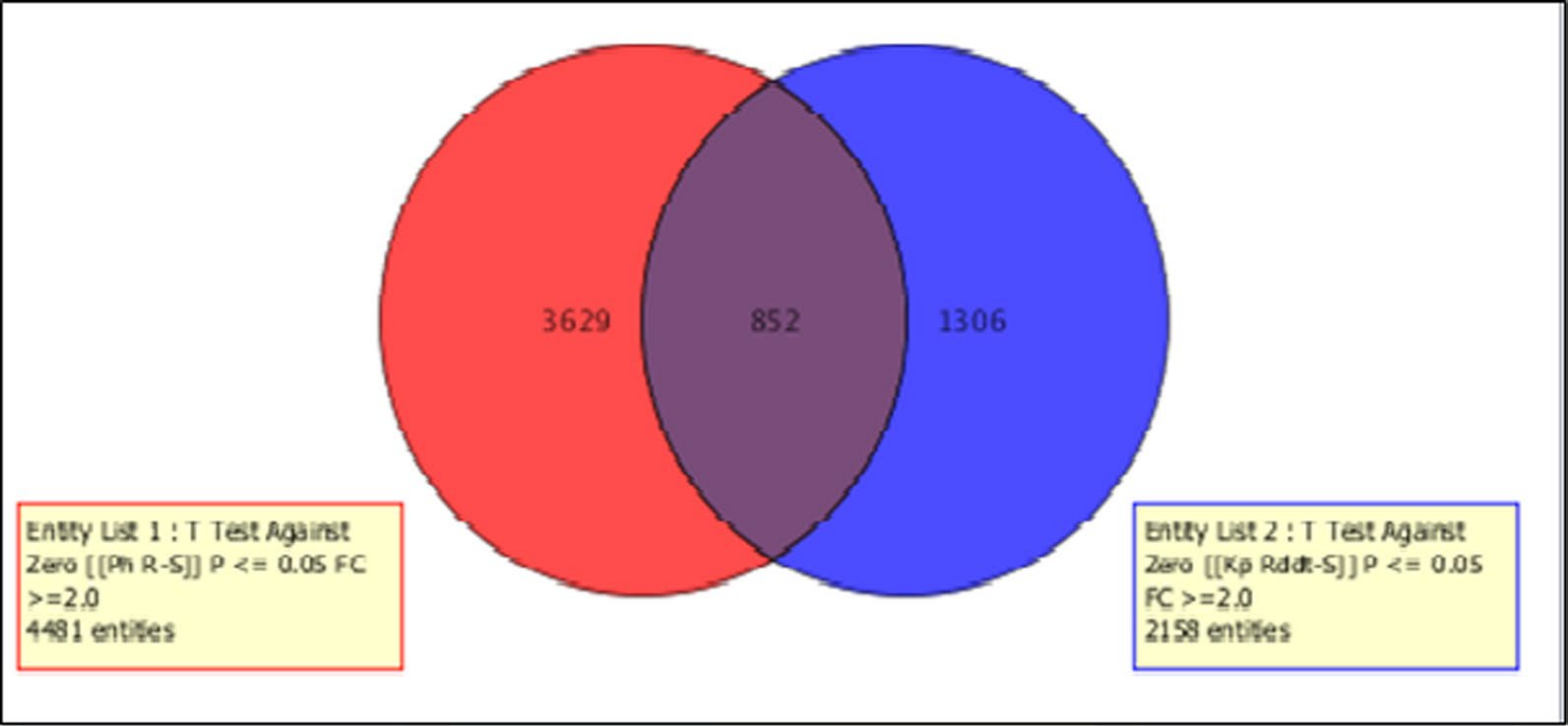
Summary of probes differentially expressed in Kpome and Pahou samples. The Venn diagrams show the number of probes significantly (P ≤ 0.05) up or down-regulated (FC ≥ 2) in each comparison as well as the commonly expressed probes

A genome-wide transcription analysis enabled us to identify the set of genes associated with DDT resistance in Pahou (Benin) and Burkina-Faso (Table 2; Fig. 2). These results were compared to Kpome (Benin) (Table 3; Fig. 3) results where high level of DDT resistance were recorded recently [15]. A total of 6610 probes were differentially expressed (FC ≥ 2 at P < 0.05) between the DDT-resistant samples from Pahou and the susceptible strain FANG with 4637 up regulated and 1973 down regulated. The comparison between the control wild type samples (Control) from Pahou and the susceptible strain FANG showed 9756 probes differentially expressed with 7489 up regulated and 2267 down regulated. In Burkina-Faso, a total of 3602 probes were differentially expressed between the DDT-resistant samples and the susceptible FANG. When comparison was made between samples from Pahou and Burkina-Faso, 1007 probes were differentially expressed with 779 over expressed and 228 down expressed as presented in (Table 2, Fig. 2). On the other hand, samples from Pahou were also compared to those from Kpome (20) and 852 common probes were differentially expressed with 326 up regulated and 526 down regulated (Table 3; Fig. 2). The most common upregulated detoxification gene in Benin and Burkina-Faso was a glutathione S-transferase, *GSTe2* with a fold change FC of 35.6; 13.3 and 3.3 in DDT resistant samples compared to susceptible FANG respectively in Pahou, Kpome and Burkina-Faso. Due to the limited number of emerging mosquitoes, we could not perform the microarrays analysis in Tanongou (Benin). Several gene families among which the most preeminent were the cytochrome P450 genes were also over expressed. Beside cytochrome P450s, other genes belonging to multiple gene families included *alcohol* and *aldehyde dehydrogenases* were up-regulated.

**Table 2:** List of top upregulated detoxification gene in *Anopheles funestus* from Pahou and Kpome exposed and unexposed to DDT

			Pahou	Kpome			
Probe name	Systematic name		R-S	Rddt-S	Ortholog in *An. gambiae*	Description	
CUST_9227_PI426302897	Afun009227		29.2	22.2	AGAP008141-PA	Argininosuccinate lyase	
CUST_13921_PI426302897	Afun013921		27.4	17.3	AGAP006709-PA	Chymotrypsin 1	
CUST_500_PI426302897	Afun000500		17.1	36.2	NA	Glycogenin	
CUST_11037_PI426302897	Afun011037		13.1	6.5	AGAP003581-PA	Alcohol dehydrogenase	
CUST_45_PI426302897	Afun000045 (GSTE2)		12.2	13.3	AGAP009194-PA	Glutathione-s-transferase gst	
CUST_1459_PI406199769	combined_c738		10.9	14.9		Short-chain dehydrogenase	
CUST_4223_PI426302897	Afun004223		9.5	12.8	AGAP008358-PA	Cytochrome p450 4d1	
CUST_1822_PI406199769	combined_c920		9.4	11.5		Glutathione-s-transferase gst	
CUST_7769_PI426302897	Afun007769 (CYP9K1)		6.1	3.0	AGAP000818-PA	Cytochrome p450 cyp9k1	
CUST_1392_PI426302897	Afun001392		6.0	2.5	NA	Glycine dehydrogenase	
CUST_6930_PI426302897	Afun006930 (CYP6M7)		4.9	4.0	AGAP008212-PA	Cytochrome p450 6a8	
CUST_8445_PI426302897	Afun008445 (GSTE4)		4.4	4.3	AGAP009193-PA	Glutathione-s-transferase gst	
CUST_12343_PI426302897	Afun012343 (CYP4H18)		4.1	5.2	AGAP008358-PA	Cytochrome p450 4d1	
CUST_3220_PI426302897	Afun003220		4.0	10.8	AGAP002867-PA	Cytochrome p450	
CUST_7646_PI426302897	Afun007646		3.9	2.9	AGAP006225-PA	Aldehyde oxidase	
CUST_5559_PI426302897	Afun005559		3.8	3.5	AGAP008783-PA	Arginase	
CUST_7469_PI426302897	Afun007469 (CYP9J3)		2.6	2.0	AGAP012296-PA	Cytochrome p450	
CUST_8615_PI426302897	Afun008615 (CYP6AA1)		2.6	5.1	AGAP002862-PA	Cytochrome p450	
CUST_13218_PI426302897	Afun013218 (CYP315A1)		2.3	2.3	AGAP000284-PA	Cytochrome p450	
CUST_15331_PI426302897	Afun015331 (CYP307A1)		29.4	3.4	AGAP001039-PB	Cytochrome p450 307a1	
CUST_9088_PI426302897	Afun009088		4.7	9.1	AGAP004900-PA	Serine protease	
CUST_14264_PI426302897	Afun014264		3.8	2.7	AGAP003785-PE	Glucose dehydrogenase	
CUST_25_PI406199775	CYP6P9a		3.4	2.8		Cytochrome p450	
CUST_10_PI426302915	CYP6M4.seq		3.2	2.3		Cytochrome p450	
CUST_13481_PI426302897	Afun013481 (GSTE1)		2.2	2.5	AGAP009195-PA	Glutathione-s-transferase gst	
CUST_1_PI426302915	CYP6M1a.seq		2.8			Cytochrome p450	
CUST_9335_PI426302897	Afun009335 (CYP6AG1)		2.7		AGAP003343-PA	Cytochrome p450	
CUST_22_PI426302915	CYP6S2.seq		7.1			Cytochrome p450	
CUST_1097_PI406199769	combined_c557		5.1			Trypsin	
CUST_7369_PI426302897	Afun007369 (CYP6P9b)		4.4		AGAP002865-PA	Cytochrome p450	
CUST_3246_PI426302897	Afun003246		4.1		AGAP006220-PA	Aldehyde oxidase	
CUST_12197_PI426302897	Afun012197 (CYP304B1)		4.0		AGAP003066-PA	Cytochrome p450	
CUST_2464_PI406199772	CD578169.1		3.9			Trypsin	
CUST_1096_PI406199769	combined_c557		3.8			Trypsin	
CUST_12666_PI426302897	Afun012666 (CYP314A1)		3.7		AGAP002429-PA	Cytochrome p450	
CUST_5005_PI406199798	AGAP003680-RA___2R		3.4		AGAP003680-RA___2R	Abc transporter	
CUST_9522_PI426302897	Afun009522 (CYP9J3)		2.8		AGAP012292-PA	Cytochrome p450	
CUST_7722_PI426302897	Afun007722		2.7		AGAP009850-PA	Abc transporter	
CUST_27_PI426302915	CYP6Z1_rvcpl_fixed.seq		2.3			Cytochrome p450	
CUST_10_PI406199775	CYP6P1		2.2			Cytochrome p450	
CUST_9068_PI426302897	Afun009068		2.2		AGAP006948-PB	Cytochrome b561	
CUST_14535_PI426302897	Afun014535 (CYP301A1)		2.1		AGAP006082-PA	Cytochrome p450	
CUST_13288_PI426302897	Afun013288		2.0		AGAP002278-PA	Abc transporter	
CUST_12342_PI426302897	Afun012342 (CYP4H14)		2.0		AGAP008358-PA	Cytochrome p450 4d1	
CUST_13475_PI426302897	Afun013475			3.2	AGAP003582-PA	Alcohol dehydrogenase	
CUST_5448_PI426302897	Afun005448 (CYP302A1)			2.6	AGAP005992-PA	Cytochrome p450	
CUST_8823_PI426302897	Afun008823 (CYP4D15)			2.4	AGAP002418-PA	Cytochrome p450	
CUST_7301_PI426302897	Afun007301 (CYP4J5)			2.2	AGAP006048-PA	Cytochrome p450	
CUST_208_PI406199788	gb-CYP12F3			2.1		Cytochrome p450	
CUST_10630_PI426302897	Afun010630			2.1	AGAP002866-PA	Cytochrome p450	
CUST_11942_PI426302897	Afun011942				AGAP011509-PA	Carboxylesterase	
CUST_3489_PI406199769	combined_c1762					Abc transporter	
CUST_8026_PI426302897	Afun008026				AGAP003578-PA	Aldehyde dehydrogenase

**Table 3:** Summary statistics for polymorphism *GSTe2* gene in F0 *An. funestus* from five localities

	N (2n)	S	π	k	h	hd	D	D*
Kpome (Benin)	18	0	0	–	1	0	–	–
Pahou (Benin)	48	5	0.00192	1.41844	2	0.284	0.63 ns	1.10 ns
Doukonta (Benin)	18	6	0.00103	0.76471	3	0.307	− 1.84 s	− 2.14 ns
Akaka Remo (Nigeria)	12	8	0.00246	1.81818	5	0.576	− 1.25 ns	− 1.48 ns
Tanongou (Benin)	4	4	0.00361	2.66667	2	0.667	2.08 ns	2.08 s
Burkina-Faso	4	2	0.00180	1.33333	2	0.667	1.89 ns	1.89 ns

### Validation of the microarray upregulation with qRT-PCR

Transcription analysis of the candidate resistance genes *GSTe2*, *CYP6P9a* and *CYP6P9b* revealed that these genes are significantly upregulated in *An. funestus* from Pahou and Kpome. Indeed the *GSTe2* was the most upregulated gene with a fold-change FC of 44.8 [12] in Pahou and 16.2 in Kpome [20] and this expression pattern goes with DDT resistance observed in both localities. The two P450 duplicated genes *CYP6P9a* and *CYP6P9b* were also upregulated with a FC of 2.9; 7.1 and 3.7; 3.4, respectively in Pahou and Kpome [12, 20].

### Correlation between the L119F mutation and DDT resistance

The genotyping of the GSTe2-L119F mutation in Kpome, and Doukonta in the southern Benin where high resistance was observed against DDT revealed a high frequency of 96% and 93% of the 119F in these locations compared to Tanongou in the North Benin (35%) where moderate resistance was observed to DDT as reported by Djouaka et al*.* [16]. Also, similar results were reported in Burkina Faso with 25% of the 119F mutation in correlation with the prevalence of DDT resistance [12].

### Role of the GSTe2-L119F mutation in DDT resistance and genetic diversity of Gste2 gene

**Fig. 4:**
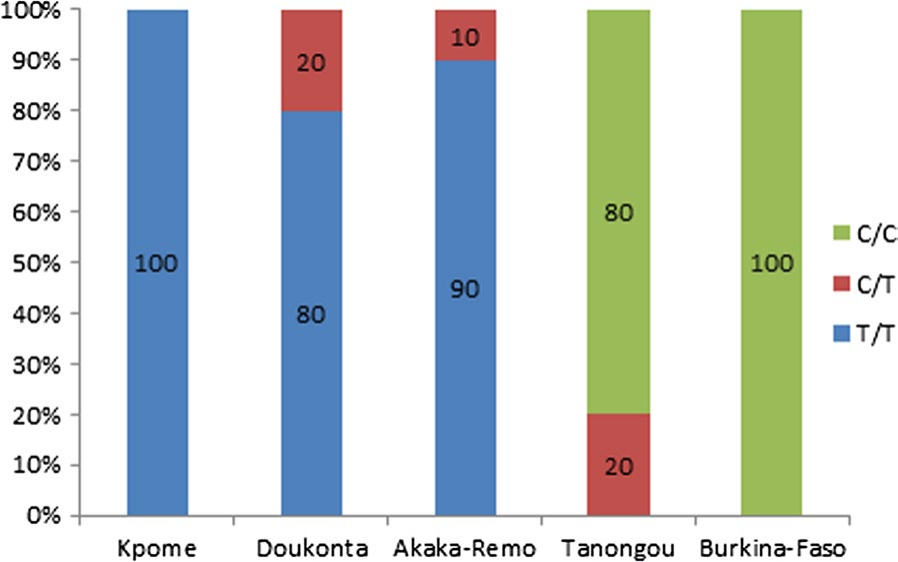
Correlation between L119F genotypes and DDT resistance in five localities

**Fig. 5:**
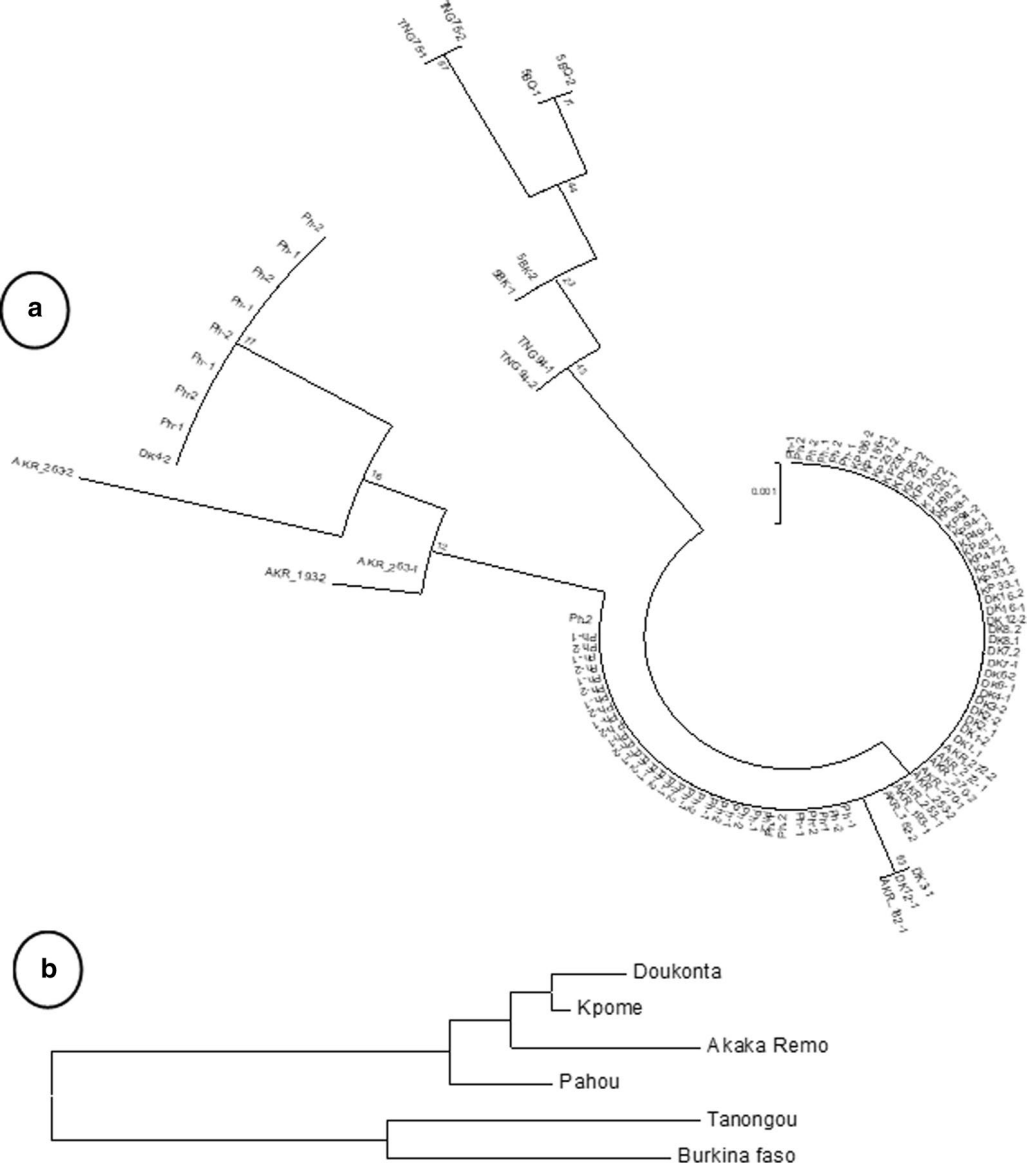
GSTe2 polymorphism in *Anopheles funestus* from 6 localities. (**a**) Maximum likelihood tree of GSTe2 in the 6 localities, (**b**) Neighbour joining tree of the *GSTe2* in the 6 localities

Full length *GSTe2* (exons and introns) was successfully amplified and directly sequenced in ten samples from each locality. These localities are Kpome, Pahou, Doukonta in the southern Benin, Tanongou in the northern Benin, Akaka-Remo in the southern Nigeria and Burkina-Faso. The L119F-GSTe2 mutation is the replacement of leucine (CTT) with phenylalanine (TTT) at the position 119. The C/C is the homozygote susceptible wild type, the T/T is the homozygote mutant genotype while the C/T is a codominant genotype. Interestingly, no T/T genotype (the homozygous resistant allele) was detected in Tanongou (North Benin) and Burkina Faso population where moderate resistance was recorded against DDT while in others populations highly resistant to DDT were almost all homozygote T/T (Fig. 4). The alignment of 739 bp of the sequenced samples showed a heterogeneity between the *An. funestus* population analysed as reported in Table 4. The analysis of maximum likehood phylogenetic tree of *GSTe2* indicated that *An. funestus* populations are structured according to their pattern of DDT resistance. The ML tree shows that sequences from southern Benin cluster closer to those from southern Nigeria where high resistance level was recorded and sequences from Tanongou cluster with those from Burkina-Faso where moderate resistance level was observed (Fig. 5a). This pattern is also supported by the neighbour-joining tree with genetic distances based on Fst estimates (proportion of the total genetic variance contained in a subpopulation) (Fig. 5b). This result suggest the presence of barriers to gene flow that are affecting the spread of resistance genes. In addition, the presence of a large indel in the *GSTe2* gene was noticed in the samples from Tanongou and Burkina Faso, but was not present in southern mosquito populations. This requires further investigation.

**Table 4:** Summary statistics for polymorphism *GSTe2* gene in F0 *An. funestus* from five localities

	N (2n)	s	π	k	h	hd	D	D*
Kpome (Benin)	18	0	0	-	1	0	-	-
Pahou (Benin)	48	*5*	0.00192	1.4 1844	2	0.284	0.63 ns	1.10 ns
Doukonta (Benin)	18	6	0.00103	0.76471	3	0.307	− 1.84 s	− 2.14 ns
Akaka Remo (Nigeria)	12	8	0.00246	1.8 18 18	*5*	0.576	− 125 ns	− 1.48 ns
Tanongou (Bruin)	4	4	0.00361	2.66667	2	0.667	2.08 ns	2.08 s
Burkina-Faso	4	2	0.00180	1.33333	2	0.667	1.89 ns	1.89 ns

## Discussion

Insecticide resistance is a complex trait and factors involved vary depending on species, insecticide and population. This research was designed to assess the underlying molecular basis driving DDT resistance in the South-North transect of Benin to improve performances of malaria controls tools.

Physiological resistance to insecticides often involves either mutations in the insecticide target site (target-site resistance), or elevated activity of detoxifying enzymes that metabolise and/or sequester insecticides (metabolic resistance). In the absence of knockdown resistance mutations in the voltage-gate sodium channel in *An. funestus* [10, 11, 14], this study showed how DDT resistance in this mosquito species is a result of both target-site resistance and up-regulation of a DDT detoxifying enzyme. Overall, this study has revealed *GSTe2* gene as a key gene implicated in DDT as a result of elevated expression rather than allelic variation through the GSTe2-L119F mutation. This is in line with previous studies [2–7, 11, 12] showing that over transcription of the GSTe2 gene confers DDT resistance and cross-resistance to permethrin. It has also been shown that the overexpression of *GSTe2* gene in DDT resistant strain of *An. gambiae* [8, 25] and the overproduction of this gene is very efficient at metabolizing DDT [26]. Also, the *GSTe2* gene has been implicated in DDT resistance in *Aedes aegypti* species from Thailand [27] showing the important role of the up-regulation of this gene in DDT resistance. Such observations are in accordance with the resistance profile of Pahou and Kpome *An. funestus* populations, which are highly resistant to DDT compared to Tanongou closer to Burkina-Faso where moderate resistance was recorded. Beside the overexpression of the *GSTe2* gene, it is acknowledged that the presence of L119F-*GSTe2* mutation confers DDT resistance in *An. funestus s.s.* populations in West/Central and East Africa [12] and this is in line with the high allelic frequency of this mutation recorded in Kpome and Doukonta. High allelic frequency of the GSTe2-L119F mutation and up-regulation of the *GSTe2* gene were observed in Kpome, Pahou and Doukonta while low regulation and low allelic frequency of the mutation were observed in Tanongou and Burkina-Faso in correlation with DDT profile observed. The consistent difference observed for this gene between the population of southern Benin (Kpome, Pahou and Doukonta) and that of Tanongou (North Benin) and Burkina-Faso suggest that possible barriers to gene flow exist between these populations. These barriers to gene flow could be due to geographic distance, because gene flow can be restricted by physical barriers separating the populations. Clarke [28] and Duke et al. [29]*.* also reported that habitat discontinuities may present barriers to gene flow. Furthermore, the genetic and behavioural divergence may be related to differences in the scale of vector control interventions between the regions or an effect of climate change could also explain this phenomenon. Such anti-vector interventions have been found to impact population size of vector populations [30]. Insecticide resistance in vector populations has been widespread with large scale exposure resulting in altered abundance, behavioural shifts and general ecology of major vector populations (e.g. *An. funestus*, *An. gambiae*) [31]. However, this observation needs to be confirmed in future studies.

Analysis of the full-length *GSTe2* gene shows a possible association between the *GSTe2* polymorphism and observed DDT resistance in the 6 localities. The 119F resistant allele is fixed in highly DDT-resistant Benin mosquitoes especially in Pahou, Kpome and Doukonta and in Nigeria (Akaka Remo), but very low in moderate resistant mosquitoes in Tanongou and Burkina-Faso showing the key role of this mutation in DDT resistance as reported by [12]. This study revealed that southern Benin and Nigeria populations of *An. funestus* are more genetically differentiated as they form a unique cluster compared to North populations. This pattern of genetic diversity of the *GSTe2* gene observed in this study support the contrast in resistance patterns between populations of *An. funestus*. In addition, a significant shift in the over-expression profile of this gene was detected across a South/North transect of Benin in line with the DDT resistance profile observed, showing that the L119F-GSTe2 mutation coupled with up-regulation of this gene confer a high level DDT resistance in *An. funestus* [12]. The consistent differences between the *An. funestus* population across Benin is likely to impact the design and implementation of resistance management strategies in Benin.

## Conclusion

Effective management of resistance requires an understanding of the dynamics and mechanisms driving resistance. This study shows that molecular basis of DDT resistance in southern Benin *An. funestus* is associated with L119F-GSTe2 mutation and over-expression of this gene. The variations observed between southern and northern populations of *An. funestus* could suggest the presence of barriers to gene flow that are affecting the spread of resistance and associated genes.

## Data Availability

All data generated or analysed during this study are included in the manuscript and its additional file.

## Abbreviations

DDT: Dichlorodiphenyltrichloroethane
qRT-PCR: Quantitative reverse transcriptase polymerase chain reaction
ML: Maximum Likehood phylogenetic tree
